# Mental Health–Related Behaviors and Discussions Among Young Adults: Analysis and Classification

**DOI:** 10.2196/17224

**Published:** 2020-05-29

**Authors:** Ryan Rivas, Moloud Shahbazi, Renee Garett, Vagelis Hristidis, Sean Young

**Affiliations:** 1 Department of Computer Science and Engineering University of California, Riverside Riverside, CA United States; 2 ElevateU Los Angeles, CA United States; 3 University of California Institute for Prediction Technology University of California, Irvine Irvine, CA United States

**Keywords:** social media, data analysis, supervised machine learning, universities, students

## Abstract

**Background:**

There have been recurring reports of web-based harassment and abuse among adolescents and young adults through anonymous social networks.

**Objective:**

This study aimed to explore discussions on the popular anonymous social network Yik Yak related to social and mental health messaging behaviors among college students, including cyberbullying, to provide insights into mental health behaviors on college campuses.

**Methods:**

From April 6, 2016, to May 7, 2016, we collected anonymous conversations posted on Yik Yak at 19 universities in 4 different states and performed statistical analyses and text classification experiments on a subset of these messages.

**Results:**

We found that prosocial messages were 5.23 times more prevalent than bullying messages. The frequency of cyberbullying messages was positively associated with messages seeking emotional help. We found significant geographic variation in the frequency of messages offering supportive vs bullying messages. Across campuses, bullying and political discussions were positively associated. We also achieved a balanced accuracy of over 0.75 for most messaging behaviors and topics with a support vector machine classifier.

**Conclusions:**

Our results show that messages containing data about students’ mental health–related attitudes and behaviors are prevalent on anonymous social networks, suggesting that these data can be mined for real-time analysis. This information can be used in education and health care services to better engage with students, provide insight into conversations that lead to cyberbullying, and reach out to students who need support.

## Introduction

### Background

The transition from high school to college marks the beginning of an important period of psychosocial development. The academic and social demands of college life are often rigorous and can pose a risk to undergraduate students’ health and well-being [1]. One example of the challenges they face is poor sleep [2], which has been linked to a number of adverse consequences, including higher rates of depressive symptoms and stress [3,4], weight gain [5], and poor academic performance [6]. Another concern for undergraduate students that has arisen in recent years is their social media use, as studies show a link between cyberbullying and major health problems such as substance use, depression, poor sleep, and suicide [7-9]. Given the array of health risks faced by undergraduate students, it is important to be aware of students’ health and risk-related behaviors to be able to provide adequate services and support, such as from psychological and medical campus services.

Traditionally, methods for monitoring the health of a population, for example, students on a college campus, have focused on case reports and surveys [10,11]. Although these methods can offer insights into health-related attitudes and behaviors, they can be time- and cost-intensive to implement. However, researchers using social media data can collect and analyze behavior data in real time [10,11], allowing health authorities to address student needs in a flexible and timely manner.

To explore the feasibility of using social media platforms to identify and predict health-related events, Young et al [12] screened geolocated Twitter messages for keywords that suggested HIV risk behaviors. The authors used negative binomial regression analyses to determine the association between tweets about HIV risk behaviors and county-level HIV data in the United States. They ran analyses to determine the association between tweets about HIV risk behaviors and county-level HIV data in the United States. The results showed a strong association between tweets about HIV risk behaviors and actual county HIV data. Additionally, De Choudhury et al [13] successfully used tweets to predict the onset of major depressive disorder with 70% accuracy. They selected tweets based on indicators such as linguistic style, use of terms associated with depression, and social network characteristics.

Yik Yak was an anonymous web-based bulletin board for users within the same geographic area (eg, college campuses) that debuted in 2013 [14]. At the time of this study, it was a popular social network for college students but faced substantial criticism. Critics argued, aided by anecdotal evidence relayed through media reports, that anonymous posting encourages harassment and bullying [14-17]. In a recent content analysis of Yik Yak conversations [18], there was no evidence of a pervasive culture of harassment and abuse. However, contradictory to this analysis, researchers have observed derogatory and incendiary comments, arguably racist and sexist messages, and several likely instances of bullying [18]. Furthermore, other research has shown that harassment is prevalent among users of Yik Yak and other anonymous social networks in Bangladesh [19]. Although Yik Yak is now defunct, the rising popularity of anonymous social networks [20] suggests that its data can still provide useful insights.

### Study Overview

In this study, we explored two types of messages students made on Yik Yak. The first type consists of posts exhibiting messaging behaviors that can have an impact on students’ health in relation to cyberbullying. This includes cyberbullying itself, which has previously been linked to health problems [7-9]. It also includes prosocial messages, which are messages sent by a user with the intention of benefiting one or more other users [21], or with the intention of seeking such messages. The prosocial messaging behaviors we selected are related to bullying and its effects on health. Two of these are seeking and offering support, as students with high depression or anxiety often turn to social media for social support [22]. The second type consists of messages that discuss one of 4 topics frequently discussed by students on Yik Yak, such as relationships and living on campus, to provide additional context to the messaging behaviors we analyzed in this study. We analyze these messaging behaviors and topics by determining which ones are most frequently discussed and which are the most popular (in terms of votes) and by finding correlations between different messaging behaviors and topics.

Our goal is to provide insights for school administrators, public health researchers, and health care professionals regarding the prevalence of messaging behaviors, such as bullying and social support, and knowledge of general topics discussed in the network. Specifically, the purpose of this study is to show that messaging behaviors that can have an impact on students’ health occur frequently on anonymous social networks, demonstrate how they are regarded by other students by analyzing their popularity, describe the prevalence and popularity of topics that are commonly discussed by college students, and explore the intercorrelations between these messaging behaviors and topics. Knowledge of these activities on anonymous social networks can inform interventions that promote healthy and prosocial behaviors among adolescents and young adults.

We also investigated the feasibility of automatic classification of messaging behaviors and topics in this study. This involved training 3 machine learning algorithms with several combinations of hyperparameters to determine the best combination for each messaging behavior and topic. We report the results of these models on test data to demonstrate their effectiveness. An accurate classification model can complement the insights provided by this study by providing administrators, researchers, and health care professionals with a tool to more easily find relevant messages.

## Methods

### Data

From April 6, 2016, to May 7, 2016, we collected anonymous conversations posted on the Yik Yak social network at 5 randomly selected universities located in each of the 4 most populous US states: California (CA); Florida (FL); New York (NY); and Texas (TX). To protect our analyses from the influence of a university with an exceptionally large number of messages, we calculated the number of messages from each university per capita with respect to the number of students enrolled at that university. We then flagged universities that had a number of messages per enrolled student more than 1.5 SDs above their state’s mean. This resulted in the removal of 1 university, the University of Texas at Dallas, leaving a total of 19 universities. Table 1 lists these universities, their status as either a public or a private university, their enrollment, and their ranking according to the 2017 *Wall Street Journal*/Times Higher Education College Rankings [23]. Enrollment and rankings are used as part of our analysis of the interplay between variables. For our analysis, we randomly selected 100 conversation threads from each of the universities (N=16,966 messages), with a mean of 892.95 (SD 128) messages per university. We analyzed the messages with respect to the type of messaging behavior, content, and popularity of message type and content.

**Table 1:** Characteristics of universities included in the study.

State and university		Public or private		Enrollment		Ranking	
**CA^a^**							
	California Polytechnic State University		Public		19,226		221
	CSU^b^ Chico		Public		16,535		467
	CSU Los Angeles		Public		20,353		700
	CSU San Bernardino		Public		17,167		700
	University of California, Irvine		Public		25,001		153
**FL^c^**							
	Florida International University		Public		53,525		550
	Florida State University		Public		36,575		226
	University of Central Florida		Public		59,894		445
	University of Florida		Public		36,731		56
	University of South Florida		Public		35,035		396
**NY^d^**							
	Cornell University		Private		14,706		9
	CUNY^e^ Hunter College		Public		20,582		350
	CUNY John Jay College of Criminal Justice		Public		15,845		700
	SUNY^f^ Buffalo State		Public		10,665		700
	SUNY New Paltz		Public		7756		423
**TX^g^**							
	Tarleton State University		Public		11,008		800
	Texas Tech University		Public		29,342		550
	University of Houston		Public		36,128		388
	University of Texas, Rio Grande Valley		Public		27,560^h^		N/A^i^

^a^CA: California.

^b^CSU: California State University.

^c^FL: Florida.

^d^NY: New York.

^e^CUNY: City University of New York.

^f^SUNY: State University of New York.

^g^TX: Texas.

^h^Fall 2016 enrollment for the University of Texas Rio Grande Valley [24].

^i^N/A: not applicable.

### Messaging Behaviors

Within the context of this study, we use the term *messaging behavior* to refer to the intent of a message, that is, what a user is trying to accomplish by posting a message. For each message, we determined if it displayed 1 of the 4 predefined messaging behaviors listed in Table 2. Among these is bullying, which we included in our analysis because of its effects on student health [7-9]. A message was considered to be bullying if it intended harm (ie, if the purpose of the message appeared to be to negatively impact the recipient’s mental health), was indicative of a power imbalance (eg, the message was racist or sexist), and if the sender repeatedly sent these messages [25]. We also included seeking help and offering support because of their relation to health and bullying—supportive environments can be seen as more healthy and possibly more likely to prevent or reduce bullying. Humor was included to better understand if users were intentionally bullied or trying to be humorous. A total of 2 undergraduate raters independently coded the selected messages for these 4 messaging behaviors; each message was assigned a messaging behavior only if both raters coded it as such.

Table 3 lists the range, SD, mean, and median for several characteristics of messages with the messaging behaviors defined in Table 2: message length, measured in both characters and words; the number of replies received by any message; the number of replies received by initial posts (ie, the first message in a thread); the post time for messages posted between midnight and noon (AM); and the post time for messages posted between noon and midnight (PM).

**Table 2:** Definitions of messaging behaviors included in the study.

Messaging behavior	Definition	Examples	Cohen kappa (number of agreements)
Seeking help	Seeking social support (eg, emotional support and help with problems) from other users	“I like don't know what to do with myself. Literally I have no one to talk to” “What's the easiest class to fill art requirement? I'm terrible at art”	0.48 (90)
Offering support	Giving social support to other users	“Hope everything gets resolved OP!” “You've got this!”	0.56 (86)
Bullying	Intends harm, indicative of a power imbalance, and messages are repeatedly sent [25]	“You people are disgusting” “In the words of DJ Khaled ‘congratulations you played yourself’ it's not hard to portray being a moron. It's quite sad actually”	0.00 (95)
Humor	Intends to be funny without bullying	“I predict my day based on my morning poo” “Why get thinner when you can get more dinner?”	0.48 (87)

**Table 3:** Characteristics of messages with each messaging behavior.

Characteristic			Range	Mean (SD)	Median
**Seeking help**					
	**Message length**				
		Characters	11-204	74.10 (47.82)	61
		Words	2-42	14.61 (9.60)	12
	**Number of replies**				
		All posts	0-50	4.14 (7.03)	2
		Initial post	0-50	5.47 (7.61)	3
	**Post time**				
		AM	12:01 AM-11:48 AM	3:38 (2:56)	2:43
		PM	12:06 PM-11:57 PM	7:33 (3:13)	8:06
**Offering support**					
	**Message length**				
		Characters	2-200	74.87 (58.39)	58
		Words	1-43	14.39 (11.25)	11
	**Number of replies**				
		All posts	0-17	0.04 (0.66)	0
		Initial post	0-17	4.57 (6.50)	1
	**Post time**				
		AM	Midnight-11:57 AM	3:27 (2:43)	2:47
		PM	Noon-11:59 PM	7:44 (3:01)	8:25
**Bullying**					
	**Message length**				
		Characters	3-230	63.26 (49.64)	47
		Words	1-40	11.92 (9.32)	9
	**Number of replies**				
		All posts	0-44	0.17 (2.42)	0
		Initial post	0-44	4.07 (11.53)	1
	**Post time**				
		AM	Midnight-11:58 AM	3:37 (2:32)	3:12
		PM	12:10 PM-11:58 PM	8:38 (3:00)	9:43
**Humor**					
	**Message length**				
		Characters	2-199	32.37 (43.96)	36
		Words	1-41	6.37 (8.43)	7
	**Number of replies**				
		All posts	0-9	0.28 (1.02)	0
		Initial post	0-9	1.83 (2.02)	1
	**Post time**				
		AM	12:02 AM-11:58 AM	3:21 (2:49)	2:40
		PM	12:09 PM-23:59 PM	7:17 (3:25)	8:09

### Message Topics

We applied latent Dirichlet allocation (LDA) to the message corpus to identify themes within the message content. LDA is a common method for categorizing topics and themes [26]. Each topic, in turn, is probabilistically associated with various words. As topics are defined purely in statistical terms, the user chooses its semantic interpretation (ie, its label) based on word probabilities for the topic.

Next, we sought to identify topics in which the LDA message classifications aligned most closely with human judgment. We did this with a subset of 1200 randomly selected messages to which the LDA assigned a topic with a probability greater than 0.7. For each of these messages, a team of 3 raters decided if the LDA topic assignment was correct (ie, does the message discuss topic *X*). On the basis of these results, we selected the 4 topics with the highest classification accuracy: relationships and sex, college living, politics, and school and classes.

In the final step, 2 undergraduate raters independently applied the 4-topic classification scheme to 96 randomly selected messages. We found that their interrater agreement was high (Cohen kappa=0.78), so all remaining messages were coded by 1 of the 2 raters. Table 4 lists Cohen kappa for each individual topic; it is undefined for politics because neither rater coded any of the 96 messages for that topic.

Table 5 lists the range, SD, mean, and median for several characteristics of messages with these topics.

**Table 4:** Cohen kappa for each topic (n=96).

Statistic	Relationships and sex	College living	Politics	School and classes
Cohen kappa	0.73	1.00	Undefined	0.77
Number of agreements	90	96	96	91

**Table 5:** Characteristics of messages with each topic.

Characteristic			Range	Mean (SD)	Median
**Relationships and sex**					
	**Message length**				
		Characters	2-252	82.18 (52.32)	70
		Words	1-47	16.17 (10.32)	14
	**Number of replies**				
		All posts	0-50	0.96 (3.43)	0
		Initial post	0-50	4.60 (6.31)	3
	**Post time**				
		AM	Midnight-11:58 AM	3:27 (2:21)	3:07
		PM	Noon-11:59 PM	8:05 (3:16)	8:55
**College living**					
	**Message length**				
		Characters	3-200	74.56 (49.98)	62
		Words	1-42	14.36 (9.52)	12
	**Number of replies**				
		All posts	0-19	0.83 (2.15)	0
		Initial post	0-19	2.60 (3.14)	2
	**Post time**				
		AM	Midnight-11:56 AM	3:34 (2:38)	2:57
		PM	Noon-11:59 PM	6:57 (3:15)	7:24
**Politics**					
	**Message length**				
		Characters	5-210	107.72 (58.43)	99
		Words	1-43	19.22 (10.65)	17
	**Number of replies**				
		All posts	0-53	0.83 (4.27)	0
		Initial post	0-53	7.13 (10.59)	4
	**Post time**				
		AM	Midnight-11:47 AM	3:26 (2:32)	3:06
		PM	12:08 PM-11:58 PM	7:52 (3:11)	7:30
**School and classes**					
	**Message length**				
		Characters	3-202	71.41 (49.59)	59
		Words	1-42	13.67 (9.38)	11
	**Number of replies**				
		All posts	0-44	0.98 (3.33)	0
		Initial post	0-44	4.39 (5.90)	3
	**Post time**				
		AM	Midnight-11:58 AM	3:41 (2:58)	2:46
		PM	12:03 PM-11:59 PM	6:58 (3:09)	7:35

### Analysis

Our analysis consisted of 3 parts: frequency of messaging behaviors and topics, popularity of messaging behaviors and topics, and interplay between variables. In the first 2 parts, we used messages that raters uniquely assigned to 1 or none of the 4 predefined messaging behaviors to assess the frequency and popularity of messaging behaviors. Similarly, we used messages that raters uniquely assigned to 1 or none of the 4 LDA-derived topics to assess the frequency and popularity of messaging behaviors. In all statistical analyses, the significance criterion was alpha=.05.

In our analysis of the relative frequencies of messaging behaviors and topics on Yik Yak, Bonferroni-corrected Fisher exact tests determined if differences in the frequencies of these messaging behaviors or topics across states were statistically significant. If we found that the differences for a messaging behavior or topic were significant, we followed this up with Bonferroni-corrected Fisher exact tests for pairwise comparisons between states of the frequency of that messaging behavior or topic.

We determined the popularity of a message by the aggregate score of +1 votes (upvotes) and −1 votes (downvotes) assigned by Yik Yak users before data collection. Notably, if a message on Yik Yak reaches a sum score of −5, it is automatically deleted from the social network. Thus, the lowest possible popularity score for a message in our dataset was −4. To protect our analyses from the influence of a few massively popular messages, we flagged messages with a score greater than 2.5 SDs above the grand mean. We then submitted the individual message scores to state × messaging behavior and state × topic analysis of variance (ANOVA), followed up by Tukey range test to further investigate any significant main effects of each ANOVA.

The third part of our analysis examined the relationship between the frequency of prosocial messages in which users sought help or offered support, the frequency of bullying messages, the popularity of these messaging behaviors, and the frequency of topics. We carried out an analysis at the university level. For each university, we calculated mean messaging behavior frequencies, the corresponding mean popularity scores, and mean topic frequencies. We measured correlations between these variables together with 2 additional variables—the number of students enrolled and school ranking.

### Classification

We conducted a series of experiments with 3 text classification algorithms on the messaging behaviors and topics in this study. The first 2 are random forest [27] and linear support vector machine (SVM) [28] classifiers with term frequency-inverse document frequency (TF-IDF) vectors [29], and the third is a convolutional neural network (CNN) text classifier [30] with global vectors for word representation (GloVe) [31].

In each experiment, we selected 1 messaging behavior or topic and regarded each message in the dataset as a tuple (*t*, *c*), where *t* is the message text concatenated with tokens for the university and state the message is from, and *c* is a class label *positive* (the selected messaging behavior or topic is present in the message) or *negative* (the messaging behavior or topic is not present). We randomly selected 10.00% (1697/16,966) of the dataset to be used as the test dataset. With the remaining training dataset, we used 5-fold cross-validation and measured the balanced accuracy [32] of each classifier to determine the best combination of classifier hyperparameters, which are then used with the full training dataset to build the final classifier model.

Table 6 lists the hyperparameters and their respective values evaluated by our experiments for each classifier. For all classifiers, we preprocess the data by removing stop words and lemmatizing the remaining words with the natural language toolkit [33]. For the random forest and SVM classifiers, we add *balanced* class weights as defined by Scikit-learn [34]. The TF-IDF vectors are also built from the implementation in Scikit-learn [34]. The remaining hyperparameters are set to their default values, as defined by the implementations of these classifiers in Scikit-learn [34]. For the CNN classifier, we perform upsampling such that the positive messages in the training data are as frequent as the negative messages and use 100-dimension GloVe vectors pretrained on Twitter data. All other CNN hyperparameters are set to their default values as defined in the code by Ng [35].

**Table 6:** Classifier hyperparameter values evaluated in our experiments.

Classifier and hyperparameter		Values
**Random forest**		
	Maximum tree depth	2, 4, 8, 16, 32, 64
	Number of trees	10, 100, 1000
**SVM^a^**		
	*C* ^b^	0.001, 0.01, 0.1, 1, 10
	Loss function	Hinge, squared hinge
**CNN^c^**		
	Filter window sizes	(2, 3, 4), (3, 4, 5), (4, 5, 6)
	Feature maps per filter window size	100, 200, 300, 400, 500, 600

^a^SVM: support vector machine.

^b^*C*: SVM regularization parameter.

^c^CNN: convolutional neural network.

## Results

### Frequency of Messaging Behaviors

A total of 11.91% (2021/16,966) of the messages were focused on 1 of the 4 predefined messaging behavior categories: seeking help, offering support, humor, and bullying. Table 7 lists the frequencies of these messaging behaviors by state. We found significant differences in the relative frequency of messages offering support (*P*<.001) and bullying messages (*P*<.001). We found no significant geographic differences for messages seeking help (*P*=.20) or for humorous messages (*P*=.40). Using separate Fisher exact tests, we found that the 2 states with the lowest rates of bullying, CA and FL, differed significantly from the states with the highest rates, NY and TX (*P*<.001 for CA vs TX and FL vs TX, *P*=.001 for CA vs NY, *P*=.003 for FL vs NY).

**Table 7:** Frequency of messaging behaviors by state.

Messaging behavior	CA^a^ (N=4496), n (%)	FL^b^ (N=4694), n (%)	NY^c^ (N=4273), n (%)	TX^d^ (N=3503), n (%)	Total (N=16,966), n (%)	Bonferroni-corrected Fisher exact *P* value
Seeking help	70 (1.56)	94 (2.00)	65 (1.52)	70 (2.00)	299 (1.76)	.20
Offering support	183 (4.07)	381 (8.12)	234 (5.48)	88 (2.51)	886 (5.22)	<.001
Bullying	61 (1.36)	68 (1.45)	98 (2.29)	93 (2.65)	320 (1.96)	<.001
Humor	140 (3.11)	134 (2.85)	144 (3.37)	98 (2.80)	516 (3.15)	.40

^a^CA: California.

^b^FL: Florida.

^c^NY: New York.

^d^TX: Texas.

We also evaluated a sample of messages that were not assigned any of the 4 predefined messaging behavior categories to better understand the nature of messaging behavior outside of these categories. This sample consisted of 100 messages that were the first messages in their respective conversation threads. We found that the majority of these messages (68/100) were commentary, for example, anticipation of future events (“Cant wait for summer!!! #summer16”), reactions to personal experiences (“I hate when people tell me to put on headphones.”), and observations (“So many economics majors on yikyak nowadays”). Other messages (16/100) asked questions that did not seek social support, for example, soliciting opinions (“Do you think all pedophiles should be executed or do you think they deserve a 2nd chance and then should be executed if they relapse?”) and polling (“Quick poll. What's your ethnicity?”). Further messages (12/100) sought people to meet with or talk to for purposes other than social support, for example, for dating (“Any cute girls in the dorms? Drop your snapchat names”) or classes (“Anyone in geology 210 on M for 4:00-5:50?”).

The remaining messages in the sample (4/100) lacked sufficient context to judge their messaging behavior. Although these broadly defined messaging behaviors are not directly related to this study and, thus, not subjected to further analysis, this sample of posts shows that future work focusing on the commentary present on an anonymous social network would likely have substantial coverage of the message content of that network.

### Frequency of Topics

Using only messages with 1 or none of the 4 LDA-derived topics (relationships and sex, college living, politics, and school and classes), we excluded 0.69% (117/16,966) of the messages from the frequency analysis. A total of 26.33% (4437/16,849) of the remaining messages dealt with either relationships and sex (2516/16,849, 14.93%), college living (644/16,849, 3.82%), politics (607/16,849, 3.60%), or school and classes (670/16,849, 3.98%). In Table 8, we break these numbers down further by state. Using separate Fisher exact tests, we found significant regional differences for each topic. NY had the fewest relationship messages and differed significantly from CA (*P*<.001) and TX (*P*=.048).

**Table 8:** Frequency of topics by state.

Topics	CA^a^ (N=4443), n (%)	FL^b^ (N=4668), n (%)	NY^c^ (N=4253), n (%)	TX^d^ (N=3485), n (%)	Total (N=16,849), n (%)	Bonferroni-corrected Fisher exact *P* value
Relationships and sex	730 (16.43)	689 (14.76)	532 (13.21)	535 (15.35)	2516 (14.93)	<.001
College living	224 (5.04)	83 (1.78)	157 (3.69)	180 (5.16)	644 (3.82)	<.001
Politics	133 (2.99)	122 (2.61)	317 (7.45)	35 (1.00)	607 (3.60)	<.001
School and classes	208 (4.68)	114 (2.44)	150 (3.53)	198 (5.68)	670 (3.98)	<.001

^a^CA: California.

^b^FL: Florida.

^c^NY: New York.

^d^TX: Texas.

We followed up on these significant effects with Bonferroni-corrected Fisher exact tests for all pairwise comparisons between states for each topic. We found significant differences in the number of college living messages between all states (*P*<.001), except for CA and TX, the 2 states with the most college living messages (*P*=.76). Finally, we found significant differences in the frequency of school-related messages between states (*P*<.001); CA and TX, where school was discussed the most, had the least significant difference (*P*=.04).

### Popularity of Messaging Behaviors

In this and the following section, we report findings on the popularity of the different messaging behaviors and topics, based on the aggregate of +1 votes (upvotes) and −1 votes (downvotes) each message elicited from Yik Yak users. We identified 1.80% (305/16,966) of the messages as popularity outliers and excluded these from further analysis.

Table 9 displays the mean popularity scores for the 4 messaging behaviors (seeking help, offering support, bullying, and humor) at the state level (CA, FL, NY, and TX). We submitted the individual message scores to a state × messaging behavior ANOVA. Both main effects were significant: *F*_3,1940_=5.11, mean square error (MSE)=4.1, and *P*=.002 for state and *F*_3,1940_=25.85, MSE=4.1, and *P*<.001 for messaging behavior. The interaction between the 2 factors was not significant (*F*_9,1940_=0.94; MSE=4.1; *P*=.49).

**Table 9:** Popularity of messaging behaviors and topics by state.

Messaging behavior	CA^a^		FL^b^		NY^c^			TX^d^			Total		
	Mean^e^ (SE)	n	Mean (SE)	n		Mean (SE)	n		Mean (SE)	n		Mean (SE)	n
Seeking help	1.04 (0.26)	68	1.37 (0.21)	92		0.78 (0.30)	63		0.53 (0.27)	70		0.97 (0.13)	293
Offering support	1.00 (0.11)	182	0.98 (0.08)	380		1.22 (0.12)	230		0.77 (0.16)	88		1.03 (0.06)	880
Bullying	0.40 (0.32)	58	0.32 (0.17)	68		0.59 (0.23)	96		0.32 (0.18)	92		0.42 (0.11)	314
Humor	1.50 (0.20)	124	1.71 (0.22)	125		2.14 (0.27)	130		1.27 (0.20)	90		1.69 (0.12)	469

^a^CA: California.

^b^FL: Florida.

^c^NY: New York.

^d^TX: Texas.

^e^Mean: Mean message popularity scores are based on the aggregate number of upvotes (+1) and downvotes (−1) per message.

We used Tukey range test to determine which state exhibited significantly different mean popularity scores. This analysis revealed that, on average, Yik Yak messages received lower popularity scores in TX than in FL (*P*=.03) and NY (*P*<.001). Additionally, Tukey test showed that bullying messages were the least popular and differed significantly from messages seeking help (*P*=.003), messages offering support (*P*<.001), or humorous messages (*P*=.001). In contrast, humorous messages were the most popular and scored significantly higher than the other 3 message types (all *P*<.001).

### Popularity of Topics

Table 10 summarizes the mean popularity scores of messages that discussed 1 of the 4 topics identified through LDA: relationships and sex, college living, politics, or school and classes. A state (CA, FL, NY, and TX) × topic ANOVA revealed main effects of *F*_3,4293_=11.23, MSE=4.9, and *P*<.001 for state and *F*_3,4293_=7.32, MSE=4.9, and *P*<.001 for the topic as well as a significant state-by-topic interaction of *F*_9,4293_=2.52, MSE=4.9, and *P*=.007. We carried out Tukey test to further investigate the significant main effects. We found that TX, the state with the lowest popularity scores overall, differed significantly from CA (*P*<.001), FL (*P*=.03), and NY (*P*<.001). Regarding the popularity of topics, school and classes was a significantly less popular topic than relationships and sex (*P*=.002), college living (*P*=.002), and politics (*P*=.001).

**Table 10:** Popularity of topics by state.

Topic	CA^a^			FL^b^			NY^c^			TX^d^			Total	
	Mean^e^ (SE)	n	Mean (SE)		n	Mean (SE)		n	Mean (SE)		n	Mean (SE)		n
Relationships and sex	1.56 (0.09)	700	1.03 (0.08)		678	1.16 (0.10)		548	0.96 (0.08)		528	1.19 (0.05)		2454
College living	1.31 (0.15)	209	1.56 (0.26)		78	1.70 (0.23)		146	0.78 (0.14)		175	1.28 (0.09)		608
Politics	1.17 (0.21)	129	1.46 (0.24)		119	1.34 (0.14)		314	1.49 (0.43)		35	1.34 (0.10)		597
School and classes	0.84 (0.12)	197	1.09 (0.20)		114	1.08 (0.18)		145	0.43 (0.09)		194	0.82 (0.07)		650

^a^CA: California.

^b^FL: Florida.

^c^NY: New York.

^d^TX: Texas.

^e^Mean: Mean message popularity scores are based on the aggregate number of upvotes (+1) and downvotes (−1) per message.

The significant state-by-topic interaction indicates that states differ with respect to the relative popularity of topics. To identify patterns of topic popularity within each state, we conducted ANOVAs with topic as a single factor, separately for each state. These ANOVAs yielded a significant effect of topic for CA (*F*_3,1231_=5.36; MSE=5.39; *P*=.001) and TX (*F*_3,928_=5.84; MSE=3.17; *P*<.001) but not for FL (*F*_3,985_=2.41; MSE=4.91; *P*=.07) or NY (*F*_3, 1149_=2.34; MSE=5.7; *P*=.07). We followed up on the significant effects for CA and TX using Tukey test. In CA, school and classes were a less popular topic than relationships and sex (*P*<.001). In TX, messages about school and classes were less popular than messages about relationships (*P*=.002) and politics (*P*<.009).

### Interplay Between Variables

We summarize the intercorrelations between the frequency of prosocial messages in which users sought help or offered support, the frequency of bullying messages, the popularity of these messaging behaviors, the frequency of topics, and school enrollment and ranking in Table 11. These correlations are based on 19 schools, except for correlations involving the variable *ranking*, for which n=18.

We found that schools with a greater frequency of help-seeking messages also exhibited a greater frequency of messages offering support (*P*=.04). Campuses where students posted less about relationships and sex sent more messages offering support (*P*=.002). Moreover, messages offering support were more frequent at higher-ranking schools (*P*=.006). Bullying occurred more often on campuses where users posted more about politics (*P*=.048) and where messages seeking help were popular (*P*=.02). Messages offering support were more popular at campuses where students posted more about classes (*P*=.04). Finally, we found that the frequency of messages about college living was positively related to the frequency of messages about classes (*P*=.04) but negatively related to the number of enrolled students (*P*=.05). The remaining correlations in Table 9 were not statistically significant.

**Table 11:** Intercorrelations at the school level.

Variable	SH^a^	OS^b^	BU^c^	PH^d^	PS^e^	PB^f^	RS^g^	CL^h^	PO^i^	SC^j^	EN^k^	RA^l^
SH	—^m^	0.48	−0.13	−0.06	0.37	0.01	−0.35	0.01	−0.38	0.36	0.17	−0.29
OS	—^n^	—^m^	−0.33	0.16	0.00	0.05	−0.66	−0.30	0.07	−0.08	0.20	−0.62
BU	—^n^	—^n^	—^m^	0.52	0.37	−0.35	0.36	0.01	0.46	−0.07	−0.07	0.10
PH	—^n^	—^n^	—^n^	—^m^	0.37	−0.02	0.19	−0.03	0.30	−0.11	0.90	−0.21
PS	—^n^	—^n^	—^n^	—^n^	—^m^	−0.18	0.26	0.19	0.16	0.47	−0.15	−0.17
PB	—^n^	—^n^	—^n^	—^n^	—^n^	—^m^	−0.20	−0.11	0.13	0.03	−0.21	−0.08
RS	—^n^	—^n^	—^n^	—^n^	—^n^	—^n^	—^m^	0.09	−0.09	−0.02	0.09	0.29
CL	—^n^	—^n^	—^n^	—^n^	—^n^	—^n^	—^n^	—^m^	−0.14	0.47	−0.45	0.29
PO	—^n^	—^n^	—^n^	—^n^	—^n^	—^n^	—^n^	—^n^	—^m^	−0.19	−0.27	−0.35
SC	—^n^	—^n^	—^n^	—^n^	—^n^	—^n^	—^n^	—^n^	—^n^	—^m^	−0.26	−0.01
EN	—^n^	—^n^	—^n^	—^n^	—^n^	—^n^	—^n^	—^n^	—^n^	—^n^	—^m^	−0.33
RA	—^n^	—^n^	—^n^	—^n^	—^n^	—^n^	—^n^	—^n^	—^n^	—^n^	—^n^	—^m^

^a^SH: seeking help.

^b^OS: offering support.

^c^BU: bullying.

^d^PH: popularity of seeking help.

^e^PS: popularity of offering support.

^f^PB: popularity of bullying.

^g^RS: relationships and sex.

^h^CL: college living.

^i^PO: politics.

^j^SC: school and classes.

^k^EN: enrollment.

^l^RA: ranking.

^m^Cells along the diagonal represent the same variable in both row and column, thus no correlation is reported.

^n^Cells below the diagonal duplicate those above the diagonal and are left blank for clarity.

### Classification Results

Tables 12 and 13 summarize the results of our trained classifiers on the test data. As accuracy can be misleadingly high for imbalanced datasets, we also report balanced accuracy. Using this metric, we see that SVM has the best performance on 5 messaging behaviors and topics (offering support, bullying, relationships and sex, politics, and school and classes), with a balanced accuracy of over 0.75 on all but the humor dataset and an average balanced accuracy of 0.7827. CNN was the second-best performer, with the best performance on humor and college living and an average balanced accuracy of 0.7645.

**Table 12:** Messaging behavior classification results.

Metric and classifier		Seeking help	Offering support	Bullying	Humor
**Accuracy**					
	Random forest	*0.9269* ^a^	*0.8120*	*0.9299*	0.6417
	SVM^b^	0.6771	0.7501	0.9240	*0.8385*
	CNN^c^	0.9098	0.6618	0.9146	0.7195
**Balanced accuracy**					
	Random forest	*0.8575*	0.7151	0.6763	0.6392
	SVM	0.8007	*0.7514*	*0.7750*	0.6543
	CNN	0.6557	0.7313	0.7702	*0.6942*

^a^The highest accuracy and balanced accuracy achieved for each messaging behavior are italicized for emphasis.

^b^SVM: support vector machine.

^c^CNN: convolutional neural network.

**Table 13:** Topic classification results.

Metric and classifier			Relationships and sex		College living		Politics		School and classes
**Accuracy**									
	Random forest	0.8209		*0.9028* ^a^		0.8704		0.9387	
	SVM^b^	*0.8521*		0.8981		*0.9405*		*0.9499*	
	CNN^c^	0.7943		0.8533		0.9399		0.9010	
**Balanced accuracy**									
	Random forest	0.7380		0.7323		0.7775		0.7899	
	SVM	*0.8145*		0.7842		*0.8605*		*0.8212*	
	CNN	0.7902		*0.8075*		0.8524		0.8147	

^a^The highest accuracy and balanced accuracy achieved for each topic are italicized for emphasis.

^b^SVM: support vector machine.

^c^CNN: convolutional neural network.

## Discussion

### Principal Findings

Owing to the growing popularity of social media across all segments of society, researchers have a plethora of data sources from which they can derive new insights about people’s social and health-related attitudes, behaviors, and beliefs. The ability to observe social media users in near real time holds particular promise in the domain of public health and health care, where rapid detection of health-relevant events and timely intervention are essential. This study aimed to explore the prevalence of information pertaining to college students’ health and well-being contained in their conversations on an anonymous social network. To this end, we analyzed the frequency and popularity of prosocial messages and bullying messages as well as the frequency and popularity of topics discussed on the web.

In our dataset, prosocial messages (seeking help, offering support, and humor) appeared more frequently than bullying messages (1735/16,966, 10.23% vs 332/16,966, 1.96%), and there were significant regional differences in the frequency of messages associated with support or bullying. Notably, Yik Yak users attending TX colleges sent the fewest supportive messages and the most bullying messages. We should interpret this finding with caution in light of the relatively small number of messages and universities considered for our study. Nevertheless, this finding highlights a potentially problematic pattern of social media use among college students that future research may link to adverse health outcomes. Unsurprisingly, bullying messages were the least popular, and humorous messages were the most popular among Yik Yak users, independent of the state in which they lived.

To identify the topics of Yik Yak messages, we relied on statistical modeling as an alternative to the subjective classification scheme recently used by Black et al [18]. A subsequent analysis of topic prevalence revealed that relationships and sex was the most frequently discussed topic among college students. School and classes turned out to be the least popular topic, as measured by the number of upvotes and downvotes a message received. From an intervention point of view, regional differences in topic frequency and popularity matter because they offer campus representatives and health professionals clues on how to best engage a student population, both on the web and offline. Although the relative popularity of topics was similar across states, we found greater regional variation in the relative frequency of topics. For example, 7.44% (318/4273) of Yik Yak messages in the state of NY discussed politics compared with only 1.00% (35/3503) in TX, and college living was addressed in 5.60% (252/4496) of messages in CA but in only 2.28% (107/4694) of messages in FL.

With our final correlational analysis, we wanted to learn more about factors that promote prosocial web-based behaviors and prevent cyberbullying at US colleges. Several findings are worth noting. At schools where students often sought help through messages, messages offering support were also more frequent. We speculate that students may offer support in response to requests for help, but the reverse relationship is also conceivable: at schools where support is offered frequently, students may feel encouraged to ask for help. A higher prevalence of supportive messages also appears to be a characteristic of higher-ranking universities. Although the *Wall Street Journal*/Times Higher Education’s college rankings [23] do not take into account social support between students, some hidden factors that lead to a higher prevalence of social support may have also been indirectly captured by their methodology. Our observation of a positive relationship between the popularity of messages offering support and the frequency of the school and classes topic may be explained by a positive response, in the form of upvotes, to support offered to students expressing frustrations with coursework and exams. It is more difficult to interpret why messages of support were sent more often at schools where relationships and sex were discussed less frequently. This requires further investigation.

Two results speak directly to the frequency of cyberbullying on college campuses. First, there was a positive relationship between bullying and the popularity of messages seeking help. One interpretation for this finding is that students react prosocially to a higher prevalence of bullying by encouraging help-seeking behavior, although they did not appear to actually offer more support (the correlation between the frequency of supporting and bullying messages was negative and not significant). An alternative hypothesis is that certain prosocial messaging behaviors can trigger cyberbullying. Additionally, students at schools with a higher incidence of bullying frequently discussed politics. This result is unsurprising given the often-heated nature of political discussions.

Of the results regarding the frequency of messages about college living, the positive relationship with the frequency of messages about classes is understandable, given that these 2 topics reflect much of the college experience. However, messages about college living are less frequent at schools with lower enrollment rates. One possible explanation may be that smaller schools have less on-campus housing relative to the number of students, but further study is necessary to make this determination.

Our text classification experiments demonstrate the feasibility of automatic classification of the messaging behaviors and topics in this study. The balanced accuracy of the SVM classifier on the test data was reasonably high for most messaging behaviors and topics. Its worst performance was with the humor dataset, which also had the lowest balanced accuracy with the random forest classifier and the second lowest balanced accuracy with the CNN classifier. This may be because of the complexity of humor—forms of humor such as innuendo, sarcasm, and satire may be difficult for a machine learning algorithm to identify.

### Conclusions

This study has strong implications for education, public health, and broader fields of health care. Educators could use similar methods to find topics that may be engaging to students on campus. In particular, campus administrators and health service units could identify topic areas where students could engage in a campus-wide dialogue. This could also be helpful for public health professionals because it would provide insight into campus conversations that lead to bullying or hostility. Educators and clinicians could work together to foster a healthier dialogue around the subject and encourage a campus culture of reaching out to fellow students to offer support. In addition to gaining insights into conversations on college campuses, this study represents a first step in guiding research focused on anonymous social networks. The results of this study can help promote the labeling and mining of social data to help students, parents, administrators, and health care workers identify cyberbullying and design interventions to stop it.

This type of work naturally presents opportunities for computer scientists working in health services as well. Mining data from anonymous social networks can extend beyond the college campus and to the public. Computer scientists can design tools to mine and categorize public social data and help create an even farther-reaching monitoring system for educators and public health professionals [36].

The major limitations of this study include the small number of colleges and universities considered, the lack of ability to generalize as Yik Yak has closed down since this study was conducted, the modest number of Yik Yak messages per school, and the relatively small number of classifier hyperparameters evaluated. We, therefore, caution against generalizing our findings until they can be replicated with larger samples and on other anonymous social networks. The main intention of this study was to understand students’ web-based behaviors and interests from their messages on an anonymous social network and, more specifically, to garner initial insight into conditions affecting prosocial and antisocial uses of social media that could be integrated into health services. We believe that the findings reported here can be a stepping stone to further research on this topic as well as differences in health behaviors and risks communicated on anonymous social networks vs nonanonymous social networks.

## Abbreviations

ANOVA: analysis of variance
CA: California
CNN: convolutional neural network
FL: Florida
GloVe: Global vectors for word representation
LDA: latent Dirichlet allocation
MSE: mean square error
NY: New York
SVM: support vector machine
TF-IDF: term frequency-inverse document frequency
TX: Texas

## References

[1] Garett R, Liu S, Young SD (2017). A longitudinal analysis of stress among incoming college freshmen. J Am Coll Health.

[2] Vail-Smith K, Felts WM, Becker C (2009). Relationship between sleep quality and health risk behaviors in undergraduate college students. Coll Stud J.

[3] Galambos NL, Dalton AL, Maggs JL (2009). Losing sleep over it: daily variation in sleep quantity and quality in Canadian students. J Res Adolesc.

[4] Williams PG, Moroz TL (2009). Personality vulnerability to stress-related sleep disruption: pathways to adverse mental and physical health outcomes. Pers Individ Differ.

[5] Roane BM, Seifer R, Sharkey KM, van Reen E, Bond TL, Raffray T, Carskadon MA (2015). What role does sleep play in weight gain in the first semester of university?. Behav Sleep Med.

[6] Curcio G, Ferrara M, de Gennaro L (2006). Sleep loss, learning capacity and academic performance. Sleep Med Rev.

[7] Bauman S, Toomey RB, Walker JL (2013). Associations among bullying, cyberbullying, and suicide in high school students. J Adolesc.

[8] Gámez-Guadix M, Orue I, Smith PK, Calvete E (2013). Longitudinal and reciprocal relations of cyberbullying with depression, substance use, and problematic internet use among adolescents. J Adolesc Health.

[9] Wang J, Nansel TR, Iannotti RJ (2011). Cyber and traditional bullying: differential association with depression. J Adolesc Health.

[10] Young SD (2014). Behavioral insights on big data: using social media for predicting biomedical outcomes. Trends Microbiol.

[11] Young SD (2015). A 'big data' approach to HIV epidemiology and prevention. Prev Med.

[12] Young SD, Rivers C, Lewis B (2014). Methods of using real-time social media technologies for detection and remote monitoring of HIV outcomes. Prev Med.

[13] De Choudhury M, Gamon M, Counts S, Horvitz E (2013). Predicting Depression via Social Media. Proceedings of the Seventh International AAAI Conference on Weblogs and Social Media.

[14] Mahler J (2015). The New York Times.

[15] Shontell A (2015). Business Insider.

[16] Valencia N (2014). CNN.

[17] Wang G, Wang B, Wang T, Nika A, Zheng H, Zhao BY (2014). Whispers in the Dark: Analysis of an Anonymous Social Network. Proceedings of the 2014 Conference on Internet Measurement Conference.

[18] Black EW, Mezzina K, Thompson LA (2016). Anonymous social media–understanding the content and context of Yik Yak. Comput Hum Behav.

[19] Nova FF, Rifat MR, Saha P, Ahmed SI, Guha S (2019). Online Sexual Harassment Over Anonymous Social Media in Bangladesh. Proceedings of the Tenth International Conference on Information and Communication Technologies and Development.

[20] Gerhart N, Koohikamali M (2019). Social network migration and anonymity expectations: what anonymous social network apps offer. Comput Hum Behav.

[21] Batson CD, Powell AA (2003). Altruism and prosocial behavior. Handbook of Psychology.

[22] Drouin M, Reining L, Flanagan M, Carpenter M, Toscos T (2018). College students in distress: can social media be a source of social support?. Coll Stud J.

[23] (2016). Times Higher Education.

[24] (2016). The University of Texas Rio Grande Valley.

[25] Garett R, Lord LR, Young SD (2016). Associations between social media and cyberbullying: a review of the literature. Mhealth.

[26] Blei DM, Ng AY, Jordan MI (2003). Latent dirichlet allocation. J Mach Learn Res.

[27] Breiman L (2001). Random forests. Mach Learn.

[28] Cortes C, Vapnik V (1995). Support-vector networks. Mach Learn.

[29] Manning CD, Raghavan P, Schütze H (2008). Scoring, term weighting and the vector space model. Introduction to Information Retrieval.

[30] Kim Y (2014). Convolutional Neural Networks for Sentence Classification. Proceedings of the 2014 Conference on Empirical Methods in Natural Language Processin.

[31] Pennington J, Socher R, Manning CD (2014). Glove: Global Vectors for Word Representation. Proceedings of the 2014 Conference on Empirical Methods in Natural Language Processing.

[32] Brodersen KH, Ong C, Stephan KE, Buhmann JM (2010). The Balanced Accuracy and Its Posterior Distribution. Proceedings of the 20th International Conference on Pattern Recognition.

[33] Bird S, Klein E, Loper E (2020). Natural Language Processing with Python.

[34] Pedregosa F, Varoquaux G, Gramfort A, Michel V, Thirion B, Grisel O (2011). Scikit-learn: machine learning in Python. J Mach Learn Res.

[35] Ng S (2018). GitHub.

[36] Benbow N, Kirkpatrick C, Gupta A, Villamar J, Chernyshov K, Cramer N, Mena L, Mayer R, Young SD (2020). An iterative process of integrating and developing big data modeling and visualization tools in collaboration with public health officials. Sage Research Methods Cases: Medicine and Health.

